# Applying EFDC Explorer model in the Gallinas River, Mexico to estimate its assimilation capacity for water quality protection

**DOI:** 10.1038/s41598-021-92453-z

**Published:** 2021-06-22

**Authors:** Claudia Villota-López, Clemente Rodríguez-Cuevas, Franklin Torres-Bejarano, Rodolfo Cisneros-Pérez, Rodolfo Cisneros-Almazán, Carlos Couder-Castañeda

**Affiliations:** 1 Facultad de Ingeniería, Universidad Autónoma de San Luis Potosí, Av. Dr. Manuel Nava # 8 Zona Universitaria, C.P. 78290 San Luis Potosi, Mexico; 2 Departamento de Ingeniería Ambiental, Facultad de Ingenierías, Universidad de Córdoba, Carrera 6 No. 77 -305, C.P. 230002 Córdoba, Colombia; 3 Instituto Politécnico Nacional, Centro de Desarrollo Aeroespacial, Belisario Domínguez 22, Col. Centro Del Cuauhtémoc, C.P. 06610 Ciudad de México, Mexico

**Keywords:** Environmental impact, Hydrology

## Abstract

Sanitary and industrial wastewater discharged into rivers, is a general problem that occurs in most of the world and Mexico is not the exception, the main goal of this research is to determine based on simulations of pollutants concentrations, the assimilation capacity of the Gallinas River against discharges of agricultural and industrial wastewater from the cultivation and processing of sugar cane under two different hypothetical simulation scenarios, based on reproducing two well know scenarios. In sugarcane cultivation, large quantities of fertilizers are used whose main active components are based on nitrogen or phosphorus compounds, therefore, the wastewater resulting from sugarcane processing contains a high organic content from 20 to 40% of inorganic compounds, such as nitrogenous substances, organic acids, and phosphorous sulfates. For this reason, the physical–chemical variables of interest analyzed in this work are the PO$_4$ (phosphate), NO$_3$ (nitrate), and DO (dissolved oxygen). With the simulation results according to each scenery, it can be determined, that despite the continuous discharge of polluting elements, the Gallinas River has a good assimilation capacity thanks to reaeration processes that permit efficient recovery of the dissolved oxygen in the water column. Gallinas River is located in the region known as the *Huasteca Potosina*, this investigation is relevant for the region due to the River is of vital importance being the main tributary that allows socioeconomic development activities in this zone. To carry out the simulations, was used the Explorer Modeling System 8.4 (EFCD) model and was performed two samplings campaign along 15 km in the water body to calibrate the numerical model to represent the dry and wet seasons during May and September respectively named as calibration scenarios.

## Introduction

In the world around 1100 million people experience the water crisis, this means that they face problems in quality and quantity. In Mexico, the National Water Commission of Mexico, affirms that industry and agriculture are the main responsible that generate most of the water pollutants. At once, the Mexican Commission for the Knowledge and Use of Biodiversity (CONABIO) is an Inter-Ministerial Commission dedicated, among other activities, to the development, maintenance and update of the National Biodiversity Information System (SNIB), to the support of projects and studies focused on the knowledge and use of biodiversity, to advise governmental institutions and other sectors, has a national monitoring network to acquire systematic and permanent samples of the quality of national waters, however, despite the efforts, only the 5% of wastwater is treated, this is due the major water consumption is for agriculture sector, mainly due the lack of treatment plants, more common found in cities and industrial parks.

It is clear that the quality and availability of water resources is essential for the subsistence and rising living standards^1,2^. Therefore, water resource must be subject to basic sustainability criteria, in order to avoid contamination and scarcity problems, generated by the effects of anthropogenic activities, for this reason many studies have been carried out with different methodologies in order to measure the magnitude of pollution.

A computational tool, to understand the dynamics and transport in shallow water ecosystems are the Water Quality Models (WQM). WQM allow to determine the behaviour and transport of toxic substances, and of course, the reliability of the models results are strongly linked with the initial parameters, mesh setup, coefficients and parameters, also allows representing the characteristics and behavior of the relationships within the system from their corresponding predictive analytical capabilities, which are useful to define approaches and manage complex problems related to water resources, ensuring a holistic approach to understand the dynamics of pollutants in shallow tropical river ecosystems.

For Water Quality Modelling we can found many and popular numerical models, as: AQUATOX, Branched Lagrangian Transport Model (BLTM), One-Dimensional Riverine Hydrodynamic, Water Quality Model (EPD-RIV1), QUAL2Kw, Water Quality Analysis Simulation Program (WASP), Water Quality for River-Reservoir Systems (WQRRS), ROMS-ICS , MIKE Ecolab/ABM, and IberWQ. A review regarding computational models of water quality can be found in the review article by Wang et al.^3^. The literature review is pointed out that most common models are: MIKE, EFDC, and Delft 3D, widely used to simulate water environmental quality in most cases of environmental impact assessment. It is necessary to mention that some other models are developed specifically for research purposes as for example for the numerical modelling of heavy metal^4^, water flows through vegetation^5^, research about turbulence models^6^, thermal discharges^7,8^, fresh waters plumes in river-sea interaction^9^ and numerical assessment of flood risk^10^.

Among many applicable and economical solutions for water quality management is the assimilation capacity, and is considered as the ability to introduce toxic wastes to waters without having a detrimental effect on public health or ecosystems, so the assimilation capacity could be used to evaluate an acceptable contamination concentration that the river flow can handle to maintain water quality in acceptable levels^11^ .

The assimilative capacity concept was first presented at the Stockholm conference in 1972, an refers to the natural ability of waters to dilute and disperse wastes and pollution without harm to the aquatic environment. Use of the assimilative capacity concept as an environmental threshold in various environmental management processes and techniques was generally founded on the premise of developing an essential framework for the subsequent design of appropriate environmental standards and land-use regulations^12^.

In a few words, assimilation capacity is the maximum amount of a contaminant that the system can hold, regardless of the contaminant source, and could be considered as an analysis-oriented tool to control the pollution of different sources in the hydrographic basins. This concept as an environmental threshold in various processes and techniques of environmental management and it was generally based on the premise of developing an essential framework for subsequent design of environmental standards and regulations for appropriate use.

In the other hand, the numerical modeling has been a very useful tool in environmental science, since the numerical simulation is considered the third approach along with theory and experimentation to try to understand the physical and biological problems. Due numerical modelling is very good analysis tool, we use it to determine the assimilation capacity and evaluating water quality, with the simulation we can predict pollutant levels, distributions and risks^13^. Likewise, numerical modeling can provide a basis and technical support for decision-making in management of pollution control, through modeled data that seek to predict with some certainty degree the water quality^14^.

Among many different models, the EFDC Explorer 8.4 was selected, and is one of the most used models for hydrodynamic and water quality, this model has the ability to predict hydrodynamics in a three-dimensional mode, solving the momentum and free surface equation, in conjunction with the continuity and mass balance equations, has coded coupled modules of salinity, temperature, sediment and contaminants transport that can be adapted to rivers, lagoons, lakes, estuaries, reservoirs and coastal water bodies^15,16^. Additionally, it incorporates dissolved oxygen, nutrients and algae module, as fundamental parameters to define the water quality.

EFDC model has been applied in various studies in different rivers in the world. The case of the Mudan River in North China where the EFDC Explorer was used for a two-dimensional model construction to simulate pollutants transport and dispersion (COD and NH3-N) in periods covered by ice and in open water to carry out a comparative analysis^16^. Another study was conducted in the Danjiangkou reservoir in China, where was developed a eutrophication model for local management of water resources in that region^17^. More applications of the EFDC can be reviewed in^14,18–27^.

With the support of the EFDC, this research focuses on the modeling of the behavior of some of the most distinctive pollutants produced by the sugar industry that affect the water quality on the Gallinas River, located in San Luis Potosí, Mexico. The simulations were performed on phosphates, nitrates, and dissolved oxygen, to establish the concentration of these substances along the river and therefore determine the assimilation capacity. Since this river has constantly received domestic and agro-industrial wastewater discharges, which gradually have deteriorated the river water quality affecting its aquatic life, it is necessary to study the behavior of the substance concentrations under certain conditions that can occur shortly, and therefore its assimilation capacity.

For the purposes of this work, were created four scenarios, the first two scenarios $S_1$ and $S_2$, were conducted for calibration purposes, representative of the dry and the wet seasons in the study zone, this means, that they were created to reproduce known conditions and validated against field measurements, once, with both scenarios calibrated, the initial conditions of the concentrations were changed to create two hypothetical scenarios $H_1$ and $H_2$, to establish if, with these new overestimated values, the concentrations are under the criterion of water quality and therefore, within the assimilation capacity of the river.

The simulations results for four different scenarios allow us to estimate the assimilation capacity of the Gallinas River, in order to establish the optimal concentration values that accomplish the water quality standards and ecological criteria to preserve aquatic life and allowing self-recover and also determine which are the most harmful hypothetical scenarios.

## Material and methods

### Description of the study area

Gallinas River is located in an important economic area related to the sugar cane industry in Mexico, in fact, the cane industry displaced over the time other important crops such as chili and cotton, which disappeared in the late nineteenth and early twentieth centuries.

Currently, in this area, four sugar mills are located named: Plan de Ayala, San Miguel El Naranjo, Plan de San Luis and Alianza Popular; the latter contributes with the principal wastewater to the Tamasopo river which is the most important tributary of the Gallinas river^28^.

**Figure 1:**
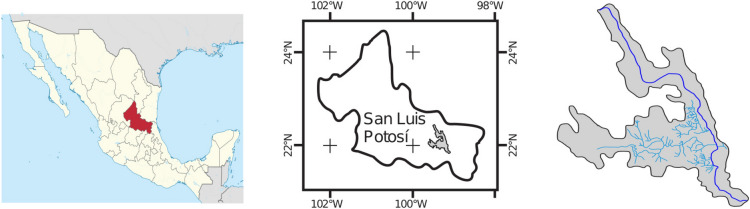
Location of the study zone. The maps were designed with Inkscape 1.0.2 (http://inkscape.org), based on OpenMaps.

Río Gallinas hydrological sub-basin has a total area of 807,568 km$^2$ and 101,127 km length, and this study focus on investigate 15 km of the Gallinas River main channel, bounded by positions (99$^\circ$ 15$^{\prime }$ W, 21$^\circ$ 59$^{\prime }$ N–99$^\circ$ 14$^{\prime }$ W, 21$^\circ$ 53$^{\prime }$ N). The two main tributaries studied that connect to Gallinas river are Piedritas stream and the Tamasopo river (see Fig. 1).

To carry out this research is necessary to monitor flow rates and water quality in these two tributaries to determine how they affect the physicochemical characteristics of the Gallinas River.

**Figure 2:**
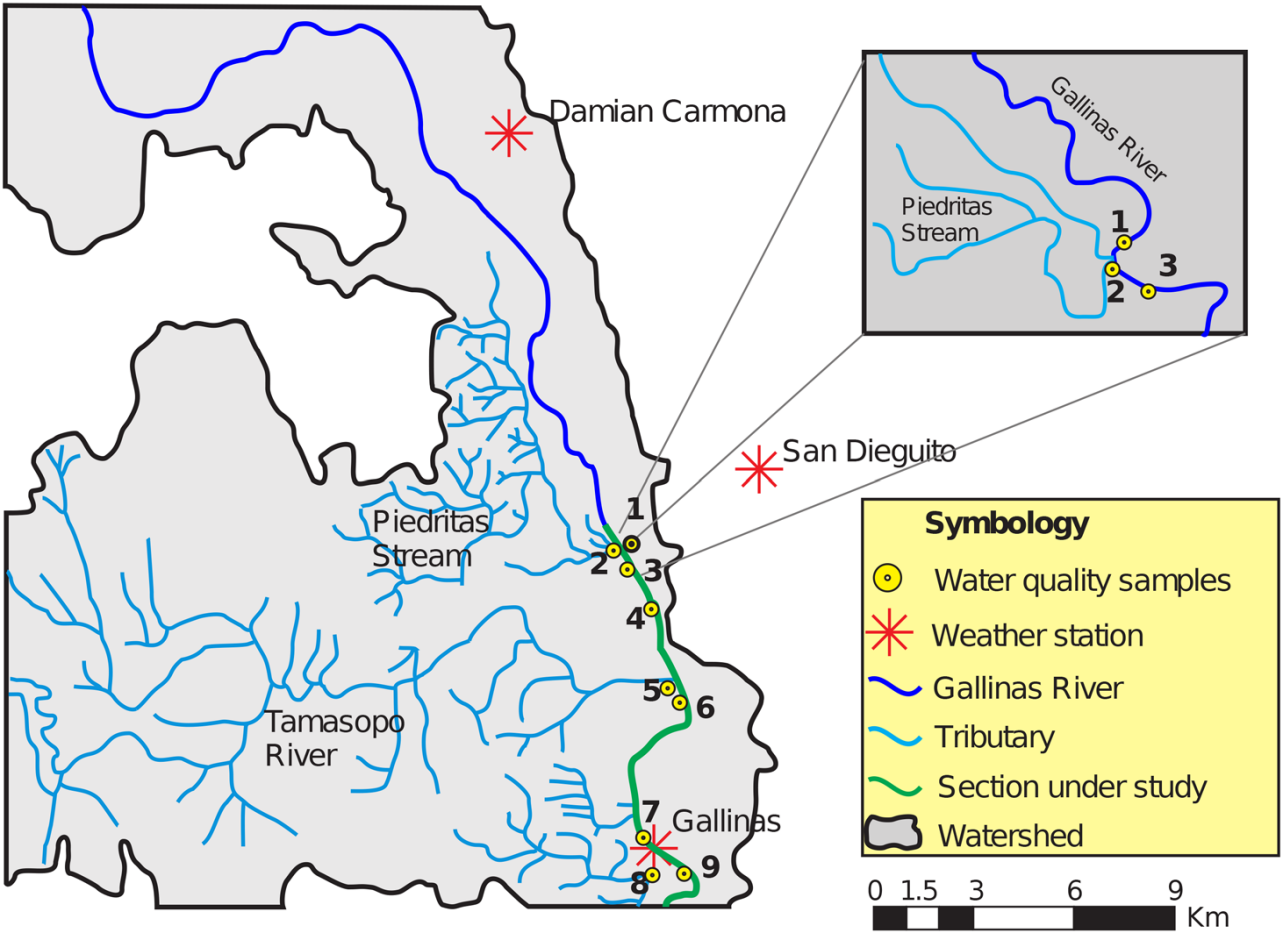
Sampling points location. The maps were designed with Inkscape 1.0.2 (http://inkscape.org), based on OpenMaps.

Nine control points were established along 15 km for water sampling, and its subsequent analysis in the laboratory, for phospathes (PO$_4$) and nitrate nitrogen (NO$_3$-N), the control points are depicted in Fig. 2.

### Hydrometeorological information

Hydrometeorological information was acquired from the National Water Commission (CONAGUA) (https://www.gob.mx/conagua), and the CLICOM system, developed by the Ensenada Center for Scientific Research and Higher Education (http://clicom-mex.cicese.mx/mapa.html), also the VARICLIM system maintained by the Coordination Center for Innovation and Application of Science and Technology (CIACyT) was used. The selected stations were Damian Carmona, Gallinas and San Dieguito (see Fig. 2).

Atmospheric conditions, forcing, temperature, precipitation, evaporation, relative humidity, and solar radiation were recorded daily and included in the model.

### Samples collection

The bathymetry used for the model was generated via measurements made using an Acoustic Doppler Current Profiler (ADCP). ADCP RiverRay (Teledyne), frequency 600 kHz, with a beam angle of 30 and cell size of 0.5 m was used for data measurement. In areas that are not measured, is required an interpolation method by taking into account various morphometric and morphologies and a wide range of scales, EFCD makes directly the interpolation using the nearest neighbor approach. Regarding the blanking zone, this is taked into account in the measurements to diminish the error^29^.

The measured velocities, the software used to process the ADCP data (in this case WinRiver II), automatically calculates by interpolation the river flow or discharge in the blanking areas. The bathymetry or measured depths were corrected by adding the blanking distance (0.3 m) to the ADCP recorded depths.

Water velocity was acquired with an accuracy of ± 2 mm/s, the river bed was estimated using a vertical acoustic beam (echo sounder) of 0.5 MHz with an embedded inclination sensor (compas/2-axis) and a temperature sensor and is operated by means of the software WinRiver II. This equipment allows us to measure flow velocities in real-time, in graphics and tabular mode. All this setup was performed for two characteristics seasons in the region: dry and wet.

Additionally, water quality parameters were measured in situ: dissolved oxygen (DO), temperature, electrical conductivity and pH, with the conductronic OX25 portable devices and the HQ40D Portable Multi Meter, both subjected to verification quality control before their use, to confirm they are working properly with the purpose to guarantee the accuracy in the parameters acquisition.

Phosphates samples were analyzed in the laboratory with the HACH 8048 method^30^ and nitrates with the HACH 8171 method.

### Numerical model

The environmental fluid dynamics code (EFDC) version 8.4 was utilized to model the assimilation capacity, the EFDC was originally developed by the United Sates Enviromental Protection Agency^31^ and was selected due to its different numerical capabilities: supports cartesian and curviliear grids, calibration test, analysis and visualization.

EFDC, is based on the continuity and velocity equations, and is one of the most widely used hydrodynamic models for shallow waters^32^, it has been widely used for modelling flow and transport processes in shallow waters bodies, like rivers, lakes, estuaries, reservoirs, wetlands, and coastal regions.

EFDC solves the 3D equations of motion and free surface equation with the Mellor-Yamada level 2.5 turbulence closure scheme. It uses stretched (or sigma) vertical coordinates and Cartesian (or curvilinear) orthogonal horizontal coordinates^33^.

For the numerical aspect, it employs a second-order accurate, three-time-level finite difference scheme with an internal–external mode splitting procedure to separate internal baroclinic mode from the external free-surface gravity wave.

The fundamental principles of the hydrodynamic model in EFDC are the conservation laws of mass, velocity, and transport equations for flows. With the basic assumption that ambient environmental flows are governed by the horizontal length scale due to its order of magnitude is greater than their vertical length scales, the formulation of the governing equations begins with the vertically hydrostatic boundary layer form of the turbulent equations of motion for incompressible flows^31^.

The EFDC governing equations are:

Velocity in *x*:

1 $$\begin{aligned} \begin{aligned} \partial _t(mHu) + \partial _x(m_yHuu) + \partial _y(m_xHvu) + \partial _z(mwu) -(mf+v\partial _xm_y-u\partial _ym_x)Hv&= -m_yH\partial _x(g\zeta +p) . \\&\quad -m_y(\partial _xh-z\partial _xH)\partial _zp+\partial _z (mH^-1A_V\partial _zu)+ Q_u. \end{aligned} \end{aligned}$$

Velocity in *y*:

2 $$\begin{aligned} \begin{aligned} \partial _t(mHu) + \partial _x(m_yHuu) + \partial _y(m_xHvu) + \partial _z(mwu) -(mf+v\partial _xm_y-u\partial _ym_x)Hv&= -m_yH\partial _x(g\zeta +p) . \\&\quad -m_y(\partial _xh-z\partial _xH)\partial _zp+ \partial _z(mH^-1A_V\partial _zu)+ Q_u. \end{aligned} \end{aligned}$$

where *u* and *v* are the velocity components (m/s) in the horizontal plane in the *x* and *y* direction respectively, $\zeta$ is the sigma coordinate, *t* is the time measured in seconds (s). $m_x$ and $m_y$ are the square roots of the diagonal components (m), $m=m_xm_y$ is the Jacobian root (m$^2$). *H* ($H=h+\zeta$), is the total depth, expressed as the sum of depth and the free surface, *p* is the pressure (m$^2$/s$^2$). $A_v$, is the vertical turbulence or turbulent viscosity (m$^2$/s). *f*, is the Coriolis parameter. $Q_u$ and $Q_y$, are the affluent–effluent movement terms (kg/m$^3$). *w*, is the vertical component velocity (m/s) and *g* the gravity acceleration.

In the left side of the Eqs. (1) and (2) the first term correspond to the temporal term (velocity change with respect to time), the second term refers to the advective component due to inertial forces, and the third is the Coriolis acceleration. On the right side the first term is the pressure force and the second represents the viscous stresses that give rise to the turbulence in the flow.

### Water quality module

The kinetic processes included in the EFDC water quality module are derived from the CE-QUAL-ICM^34^. The governing mass balance equation to transport water quality parameters is expressed as:

3 $$\begin{aligned} \begin{aligned} \partial _t(m_xm_yHC) + \partial _x(m_yHuC) + \partial _y(m_xHvC) + \partial _z(m_xm_ywC)&= -m_yH\partial _x(g\zeta +p) . \\&\quad -m_y(\partial _xh-z\partial _xH)\partial _zp+\partial _z(mH^-1A_V\partial _zu)+ m_xm_yHS_c. \end{aligned} \end{aligned}$$

where, *C* is the concentration of a water quality variable, $A_x,A_y,A_z$ correspond to the turbulent diffusivity terms in the *x*, *y*, *z* directions respectively. $S_c$ represent the sources and sinks per unit volume. *H* is the depth of the water column. $m_xm_y$ are the metrics of the curvilinear coordinates. In fact, the last three terms of the left side of the equation models the advective trasnport and the first three terms on the right side represents the diffusive transport. Finally, the last term in Eq. (3) represents the kinetic processes and external sources for each state variable. The models solves the Eq. (3) using a fractional step procedure that decouples the kinetic terms from the physical transport terms.

Next, the kinetic equations for each of the state variables studied in this research are formulated as follows:

4 $$\begin{aligned} \begin{aligned} \partial _t(PO_4P+PO_4d)&= \sum _{x=c.d.g.m}\left( FPI_x \times BM_x + FPIP_x \times PR_x - P_x \right) \times APC_x \times B_x \\&\quad + K_{DOP} \times DOP + \partial _z(WS_{TSS} \times PO_4p) + \frac{BFPO_4d}{\Delta Z} + \frac{WPO_4p}{V} + \frac{WPO_4d}{V} \end{aligned} \end{aligned}$$

where, $PO_4d+PO_4p$ equals to the total phosphate $(PO_4t)$ in g/m$^3$; $(PO_4d)$ is the dissolved phosphate (g/m$^3$); $PO_4p$ corresponds to the particulate phosphate (g/m$^3$); FPI, is the fraction of phosphorus metabolized by the algae, produced as inorganic phosphorus (dimensionless); BM, is the basal metabolic rate of the algae (day$^{-1}$); FPIP is the fraction of phosphorus produced as inorganic phosphorus (dimensionless); PR, is the precipitation rate of the algae (day$^{-1}$); P, is the production rate of the algae; APC, is the average phosphorus-carbon ratio for all algae groups (g); B, is the biomass of the algae (g/m$^3$); K$_{DOP}$, is the dissolved organic phosphorus mineralization rate (day$^{-1}$); DOP, is the concentration of organics phosphorus (g/m$^3$); WS$_{TSS}$, is the sedimentation rate of suspended solids (m/day), provided by the hydrodynamic model; $BFPO_4d$ is the sediment-water phosphate exchange flow (g/m day);$WPO_4t$, is the external loads of total phosphate (g/day); and *V* is the cell volume (m$^3$).

The kinetic equation for $NO_3-N$ is expressed as follows:

5 $$\begin{aligned} \begin{aligned} \partial _t(NO_3)&= \sum _{x=c.d.g.m}(PN_X-1) \times P_X \times ANC_X \times B_X \times APC_x \times B_x + KNIt \times NH_4 - ANDC \times Denit \times DOC\\&\quad + \frac{BFNO_3}{\Delta Z} + \frac{WNO_3}{V} \end{aligned} \end{aligned}$$

where $PN_X$, is the ammonium uptake by algae (dimensionless), $ANC_X$ is the nitrogen-carbon ratio constant in the algae (g), *KNIt* is the nitrification rate (day$^{-1}$), $NH_4$, is the ammonia nitrogen concentration (g/m$^3$), ANDC, is the mass of nitrate nitrogen reduced by mass oxidized dissolved organic carbon (g), *Denit*, is the denitrification rate (day$^{-1}$), DOC, is the dissolved organic carbon concentration (g/m$^3$), $BFNO_3$, is the sediment-water nitrate flow exchange (g/m$^2$ day), applied only to the bottom layer, and $WNO_3$ is the nitrate external charges (g/day).

The kinetic equation for *OD* is expressed as follows:

6 $$\begin{aligned} \begin{aligned} \partial _t(DO)&= \sum _{x=c.d.g.m} \left( (1+0.3(1-PN_X))P_X - (1-FCD_X)\left( \frac{DO}{KHR_x+DO} \right) BM_X \right) +\\ + AOCR(B)-(AONT)(Nit)(NH_4)-AOCR(K_{HR})(DOC)-\frac{DO}{KH_{COD}+DO} + K_{COD}(COD) \\&\quad + K_{R}(DO_S-DO) + \frac{SOD}{\Delta V} + \frac{WDO}{V} \end{aligned} \end{aligned}$$

where, $PN_X$ is the absorption of ammonia by algae (dimensionless); $P_X$, is the algae production rate (day$^{-1}$); $FCD_X$, is the basal metabolic fraction (dimensionless); *DO*, is the dissolved oxygen concentration (g/m$^3$); *KHR*, is the mean saturated oxygen saturation constant of algae (g/m$^3$); $BM_X$, is the basal algae metabolism rate (day$^{-1}$); *AOCR*, is the dissolved oxygen ratio in respiration (g); *B*, is the algae biomass; *AONT*, is the mass of dissolved oxygen consumed per unit mass of nitrified ammonium nitrogen (g); *Nit*, is the nitrification rate(day$^{-1}$); $NH_4$, is the ammoniacal nitrogen concentration (g/m$^3$); $K_{HR}$, is the heterotropic respiration rate of dissolved organic carbon (day$^{-1}$); *DOC* is the dissolved organic carbon concentration (g/m$^3$); $KH_{COD}$, is the constant saturation of the dissolved oxygen medium required for the oxidation of *COD* (g/m$^3$); *COD*, is the concentration of oxygen demand (g/m$^3$); $K_r$, is the aeration coefficient (day$^{-1}$); $DO_S$, is the saturated dissolved oxygen concentration (g/m$^3$); *SOD*, is the oxygen demand of the sediment (g/m day) and *WOD* is the extenal oxygen charges (g/day).

## Model setup

The numerical model was configured for two periods: dry and wet seasons, the dry season is established from May 1 to 30, and the wet season from September 1 to 30 in 2018.

The domain area was discretized using a curvilinear grid, and the model was set up, with the bathymetry, free water surface, bottom roughness, all the boundary conditions necessary to approximate accurately the simulations.

**Figure 3:**
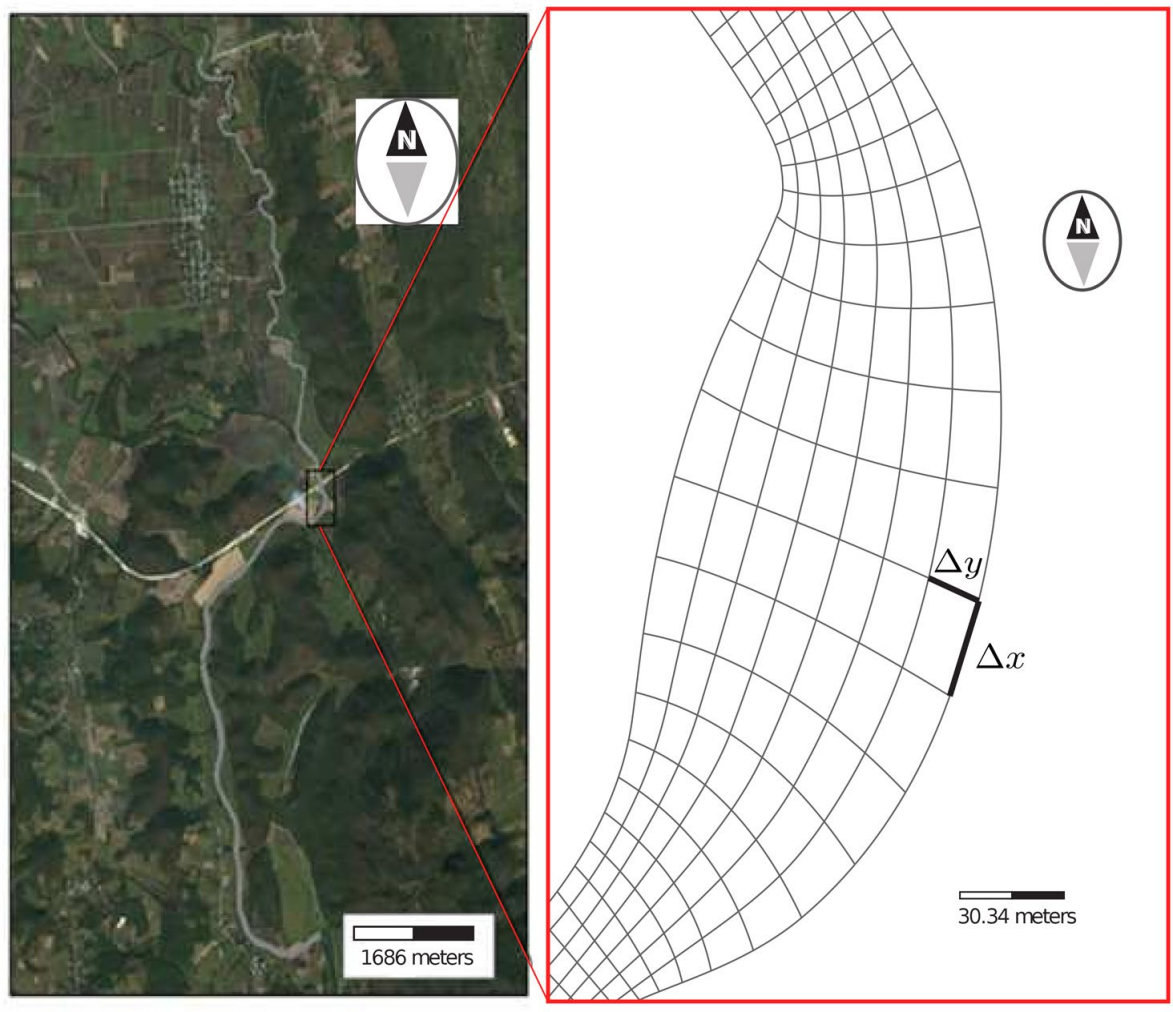
Curvilinear numerical grid generated. This figure was generated using the post-processing visualization tool of the EFDC Explorer (https://www.eemodelingsystem.com/).

The curvilinear numerical grid was generated with the Curvilinear Grid Generator for the EFDC (CVLGrid), with a $\Delta X$ that varies from 4.0 to 25.5 m and $\Delta Y$ varies from 1.9 to 21.2 m, for a total of 8666 cells (see Fig. 3). The grid fits properly the river sinuosity, in fact, is recommended keep the grid as uniform as possible to minimize possible numerical problems^35^.

For the estimation of $\Delta t$, Dynamic Timestep option is enabled, this means EFDC Explorer automatically calculates the $\Delta t$ every time step, assuring stability. In general, two options, are possible, establish, $\Delta t$ as a constant, or estimate $\Delta t$ every step in the simulations involving the small changes in velocities. Although calculate the best $\Delta t$ every time step could be computationally costly, is better. When Dynamic Timestep option is enabled, the model internally is checking the numerical stability with the Courant–Friedrichs–Lewy condition every time step.

**Figure 4:**
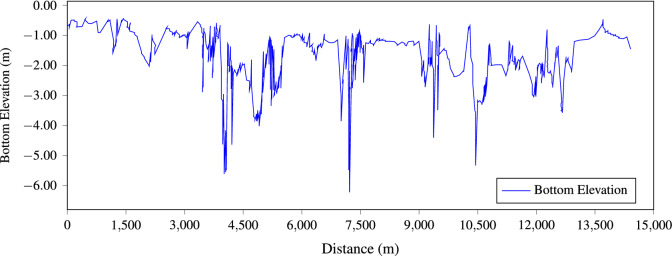
Bottom elevation.

Model Setup includes the field-collected bathymetric data depicted in Fig. 4, river depths range from 0.301 to 6.271 m).

**Figure 5:**
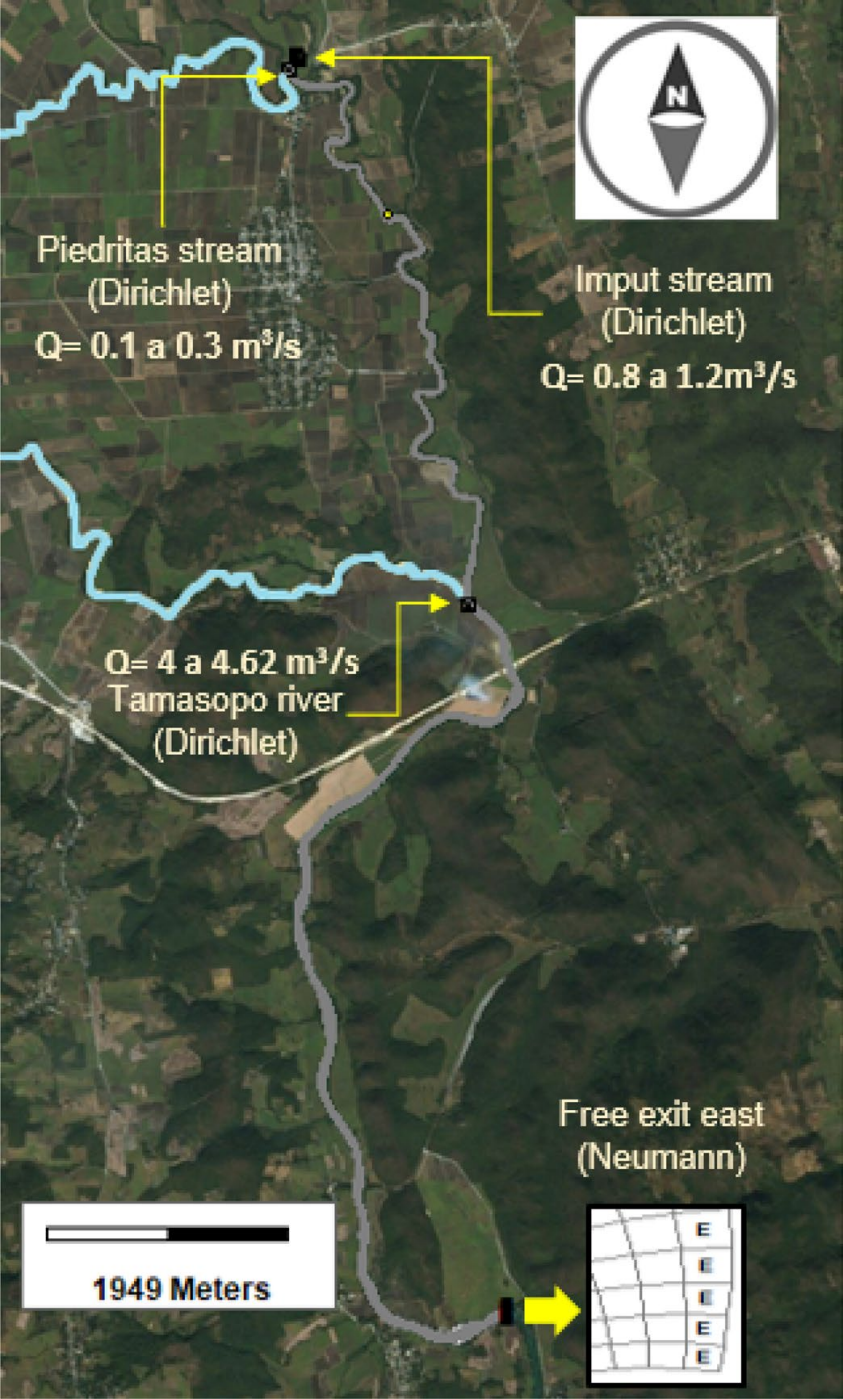
Inflow and outflow conditions, imposed in the Gallinas River. This figure was generated using the post-processing visualization tool of the EFDC Explorer (https://www.eemodelingsystem.com/).

Inflows and outflows conditions are depicted in Fig. 5, and their respective concentrations of water quality variables are showed in Table 1. On the upstream boundary, the river flow was imposed with a variation from 0.8 to 1.2 m$^3$/s, similarly, the Piedritas stream varies from 0.1 to 0.3 m$^3$/s. The Tamasopo river was established with a variable flow from 4 to 4.62 m$^3$/s, downstream an open boundary was imposed. The initial temperature was imposed at 27 $^{\circ }$C.

**Table 1:** Water quality concentration values established as inflow conditions.

Flows	Q m$^3$/s		PO$_4$-P mg/L		NO$_3$-N mg/L		OD mg/L	
	Dry	Rain	Dry	Rain	Dry	Rain	Dry	Rain
Upstream flow	0.8	1.2	0.08	0.21	1.3	0.6	6.7	7.3
Piedritas stream	0.1	0.3	0.09	0.16	1.0	0.6	6.3	7.1
Tamasopo river	4.0	4.62	0.08	0.13	1.8	0.5	7.0	7.3

**Figure 6:**
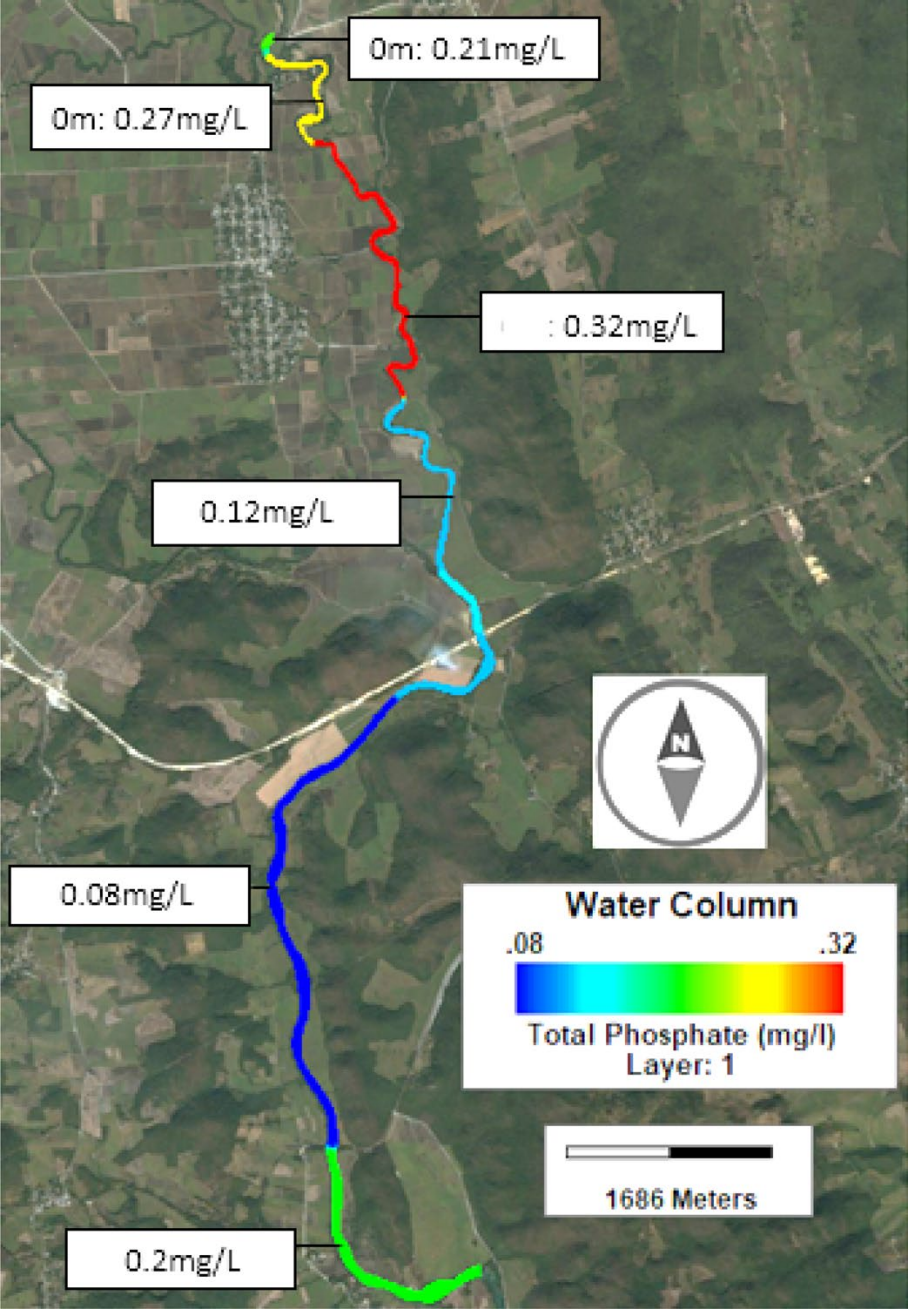
Initial concentration for the phosphates, established by zones according to the samples in the field. Similar initializations, were carried of for NO$_3$-N and DO. This figure was generated using the post-processing visualization tool of the EFDC Explorer (https://www.eemodelingsystem.com/).

The most important initialization was to define six zones along the river according to the water quality results of the selected sampling points (see Fig. 6).

Calibration is one of the most important processes in a numerical model setup since the presence of diffuse sources can hardly be represented^16^. Commonly the model parameters are adjusted by trial and error until the simulation results fit with the acquired data^17^. The adjusted parameters during the calibration process were: reaeration constant rate, rearation readjustment factor, reaeration constant temperature rate, maximum nitrification rate, reference temperature for nitrification and mininum phosphate mineralization rate. Table 2 shows the values used for the model calibration, the calibration period was 15 days, and the dynamic time step was selected to facilitate the model stability and increases computational performance. For the parametrization process, the EFDC Explorer automatically initializes the water quality parameters using reference values and they were adjusted in the calibration process, taking into account similar studies^36^ and our own expertise.

Additionally, a statistical observation was made to evaluate the precision of the simulations, regarding hydrodynamic calibration, the root of the mean square error (RMSE) was used to indicate the good or poor quality of the model. If with the RMSE is obtained a value of less than 0.3 then a good agreement is obtained, else if the value is near 1, the model has not a good fit. The RMSE is useful to compare the fit of the magnitudes and directions with respect to the measured values for both seasons, dry and wet^37^.

For water quality data, the Nash Sutcliffe efficiency coefficient (NSE) was used, which is commonly used to assess the predictive level of hydrological models, it has a threshold of $0.5<$ NSE $< 0.65$ to be satisfactory, nevertheless a value near to 1 is considered a better fit^38^.

**Table 2:** Coefficient and constants used for the water quality model.

Parameter	Value	Unit
Reaeration constant rate	5.32	–
Reaeration reset factor	1.0	–
Temperature rate of the re-airing constant	1.1	–
Maximum nitrification rate	0.07	day$^{-1}$
Reference temperature for nitrification	27	C
Minimum phospate mineralization rate	0.05	day$^{-1}$
Water quality module wet season	–	–
Reaeration constant rate	5.32	–
Reaeration reset factor	1.7	–
Temperature rate of the reaeration constan	1.0	–
Maximum nitrification rate	0.1	day$^{-1}$
Reference temperature for nitrification	27	C
Minimum phospate mineralization rate	0.05	day$^{-1}$
Horizontal eddy viscosity	0.1	m$^2$/s
Horizontal diffusivity	0.05	day$^{-1}$

**Table 3:** Formulation of the simulation scenarios.

Stage	Season	Flow	Concentration variation of Piedritras and Tamasopo flow		
			PO$_4$-P	NO$_3$-N	DO
$S_1$	Dry	Without change	Not changed	Not changed	Not changed
$S_2$	Wet	Without change	Not changed	Not changed	Not changed
$H_1$	Dry	Without change	80	300	50
$H_2$	Wet	Without change	80	300	50

**Figure 7:**
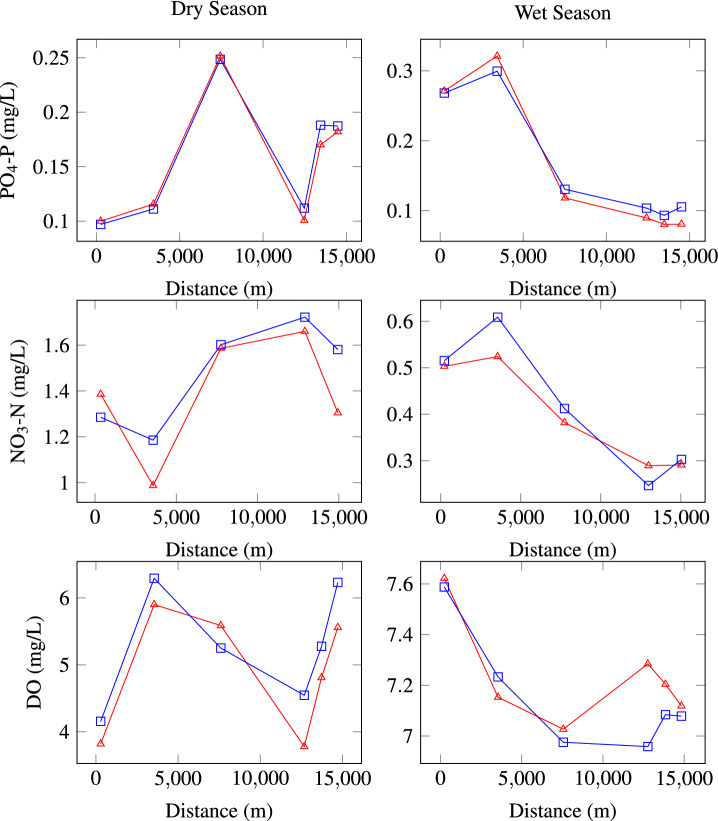
Comparison between the observed and simulated concentrations along the river for the scenarios $S_1$ and $S_2$, squares represent the results of the simulations when the hydrodynamic is steady, triangles refer to the acquired data. The fit between observed and simulated values was used to validate the parameters used in the model, and to conclude that the simulations are correctly reproducing known conditions.

### Parameter settings for the simulations of PO$_4$-P, NO$_3$-N, DO

Water quality is determined through the physical, chemical and microbiological characterization of the water and their respective comparison with the normativity standards, and ecological criteria. In Mexico these criterion are established by the National Water Commission^39^.

In Mexico the ecological the agreement CE-CCA-001^40^ establishes the ecological criteria for water quality, and the limits necessary for drinking water and protection of aquatic life are:

- Phosphates, 0.1 mg/L (for drinking water)
- Nitrates, 5 mg/L (for drinking water)
- Dissolved oxygen, 4 mg/L (for drinking water)
- Dissolved oxygen, 5 mg/L (protection of aquatic life)

For this research, the characteristic scenarios were established as $S_1$ and $S_2$, which represent dry and wet seasons respectively, these scenarios were developed for calibrations purposes and the goal is to reproduce the concentrations behaviour measured along the river, additionally two hypothetical scenarios were established named $H_1$ and $H_2$ in order to investigate if the river can continue to have assimilative capacity. For the hypothetical scenarios, the PO$_4$-P and DO are decreased by 80% and 50% respectively, for NO$_3$-N concentrations are increased by 300% for both hypothetical scenarios (see Table 3), the percentages of increase and decrease were established considering the initial values of scenarios S$_1$ and S$_2$ and the values of the water quality ecological criteria (CE-CCA-001).

These hypothetical concentrations reductions of PO$_4$-P to 80%, for the dry and wet seasons, obey the values to fulfill the CE-CCA-001 (0.1 mg/L), and NO$_3$ was increased by 300% to reach the maximum concentration criteria (5 mg/L). Although the DO results show good agreement with the ecological criteria, it is necessary to observe the river response in case the DO is reduced more than 50%.

In brief, the hypothetical scenarios were proposed in order to determine, in what percentage, the pollutants have to be reduced so that the river continues with the assimilation capacity or estimate the least favorable conditions in the presence of these pollutants.

As mentioned earlier, 15 days were used to calibrate the water quality model, a snapshot value of concentrations are shown in Fig. 7, and the corresponding Nash Sutcliffe coefficients are shown in the Table 4.

**Table 4:** Nash Sutcliffe coefficient results for the water quality calibration.

PO$_4$-P		NO$_3$-N		DO	
Dry	Wet	Dry	Wet	Dry	Wet
0.99	0.89	0.73	0.94	0.65	0.80

**Figure 8:**
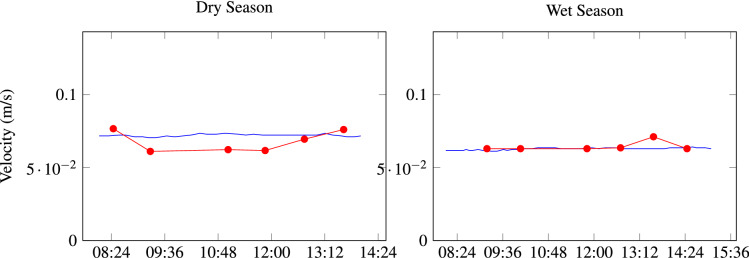
Velocity field, acquired data (circle) vs simulated data (dashed lines), for a small period of time. To calculate RMSE all the time serie was used (dry season: RMSE = 0.007; wet season: RMSE = 0.002).

## Simulation results

As previously established, the numerical simulations presented in this research were performed for two characteristic scenarios: wet and dry seasons, to calibrate the model and reproduce the conditions as accurately as possible, and two hypothetical scenarios towards predict the assimilation capacity changing pollutant concentrations.

To achieve correctly the prediction of the pollutants transport, first, is necessary a correct hydrodynamic calibration, which was obtained through the execution of multiple simulations, and was selected the one, which best fit between observed and simulated data.

The calibrations process is to repeat several simulations with different parameters until the results are according to the data acquired. To start this process, EFDC explorer automatically initializes the water quality parameters using reference values, and progressively the parameters are adjusted according similar studies^36^ and with our own expertise.

For the selected simulation, the correlation of observed and simulated hydrodynamic field (water velocity magnitude), indicates an RMSE of 0.007 and 0.002 for dry and wet seasons respectively, this means a very good fit between observed and measured data, therefore the hydrodynamic of the Gallina River were reproduced successfully, through simulations.

As an example for the fit between simulated and measured data, Fig. 8 shows the velocity calculated versus the measured during a small lapse of time in one control point, obtaining a good fit, of course, more data field were recorded and compared in all control points and for almost the 24 h of the day, nevertheless, the fit obtained is very similar, and it is not possible to show all the recordings.

**Figure 9:**
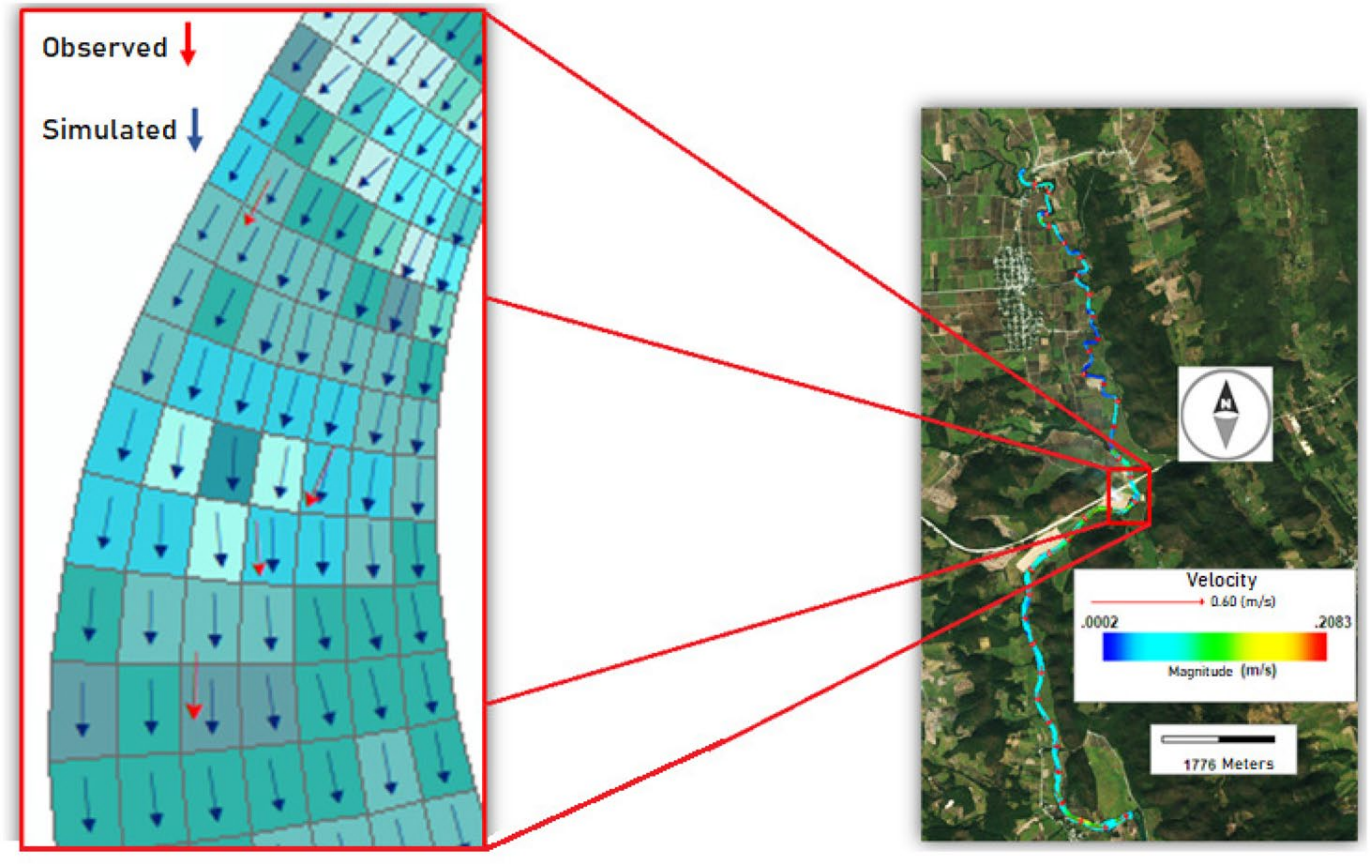
Velocity vectors, red (measured) vs blue (modeled), the fit is satisfactory in terms of magnitude and direction. This figure was generated with ArcGis 10.5 (https://desktop.arcgis.com/en/).

Figure 9 shows a comparison of observed versus modeled velocity vectors. It is necessary to mention that Gallinas river velocities are low, around 0.0002–0.2083 m/s.

The low velocities in the Gallinas River are mainly due to its hydromorphological characteristics. Typically, the longitudinal profile of a river has areas of rapids and backwaters; Fig. 4 shows that the average gradient of the Gallinas River (in the modeled section) is quite low, with the presence of deep areas or pools that are reflected in river backwaters with low velocities, and it is in the high water areas where the current gains speed. In these backwater areas with low velocities, dispersion processes are disadvantaged causing an increase in pollutant reaction rates and a decrease in dissolved oxygen available for degradation. This is clearly observed between 4.5 and 6.0 km of the modeled section, which corresponds to a deeper zone and where the decrease in oxygen occur.

For dry season, S$_1$ (normal conditions) we can state that the concentrations in the river section of Tamasopo discharge meet the ecological criteria, in the section of the confluence of Tamaposo river the model suggest concentrations of 0.19 mg/L, near to the maximum of the range that can cause eutrophication. The values simulated match with the concentrations measured in situ.

**Figure 10:**
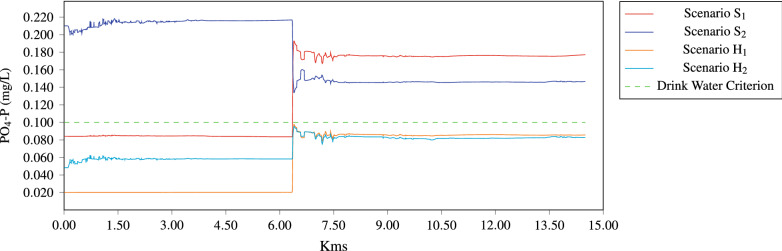
Concentration of PO$_4$-P along the river when the hydrodynamic is steady.

According to the obtained simulations results, the S$_2$ scenario (wet season) is the most unfavorable for the PO$_4$-P due exceeds the ecological criteria of 0.1 mg/L for use as drinking water supply and protection of aquatic life (CE-CCA-001/98). According with this, it can be inferred that Gallinas River has problems with the phosphate concentration because the normal values range from 0.005 to 0.02 mg as depicted in Fig. 10.

It is estimated that phosphate concentration elevation in the Gallinas River is mainly increased by sugar cane crops, due to chemical fertilizer is applied by dispersion in the zone, which makes it more feasible for the residues to reach the river since the crops are located on the banks of the water body, it is necessary to point out, that the best time to fertilize the crop is in the wet season, which explains why the concentration of phospates increase during the wet season. Must be considered that in addition to fertilizers there are also pesticides, which contain organophospate compounds that also cause harm to the environment.

Figure 10 is depicted the behavior of the phosphates concentrations for the four scenarios along the river, specifically for the hypothetical scenarios (H$_1$ dry, H$_2$ wet) the simulations demonstrate that is possible to maintain the PO$_4$-P levels below the water quality ecological criteria.

**Figure 11:**
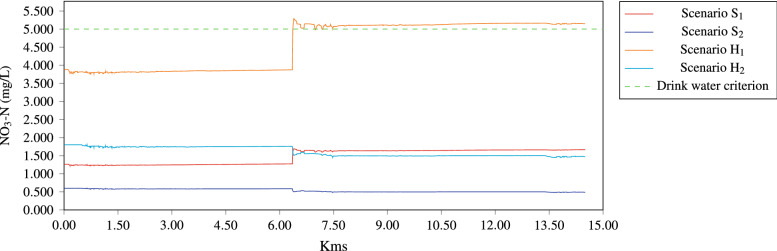
Concentration of NO$_3$-N along the river when the hydrodinamic is steady.

Regarding the NO$_3$-N, it was found that the best time for the degradation of this pollutant is in the rainy season. In Fig. 11 it can be seen the behavior of the NO$_3$-N concentration, according to the results, NO$_3$-N satisfy the ecological criteria CE-CCA-001/89 (5 mg/L for drinking water supply), therefore, simulations indicate that the assimilation capacity of NO$_3$-N in the Gallinas River is appropriate due to concentrations are below the ecological criteria for drinking water supply, being one of the fundamental uses of this water body. As for hypothetical scenarios H$_1$ and H$_2$, NO$_3$-N increased to 300% for the scenario H$_1$ (dry season), the results shows, nitrate pollution problems downstream, after the Tamasopo river contribution, therefore under this scenario the Gallinas River is not capable of assimilating the pollutant due to exceeds the water quality ecological criteria, while for H$_2$ (wet season) it still remains below the criteria for the use as a source of drinking water supply.

About the NO$_3$-N, the Official Mexican Norm-127, indicates that the maximum permissible level of water for human consumption is equivalent to 10 mg/L, which means that all proposed scenarios remain within the permissible limit.

It is worthwhile to remark, that nitrates control in water is important for human consumption, especially for children, because they can cause serious side effects, as the blue baby syndrome (methemoglobinemia), and though, nitrates are not directly harmful, it is a health hazard due to its conversion to nitrite, with hemoglobin in the blood, generating methemoglobinemia^41^. Concerning the aquatic life protection in Mexico, there is not established ecological criteria for NO$_3$, nevertheless, some research suggests that nitrate is less toxic than nitrite and ammonia, as a result of its low branchial permeability, which makes absorption through the gills more limited. The toxic action of NO$_3$ is basically due to the conversion of respiratory pigments into forms that are incapable of transporting and releasing oxygen, which means nitrate must previously be converted to nitrite under the internal conditions of the animal$^{?}$.

According to some studies, a water body is more susceptible to contamination by non-point sources, since those dominate the entry levels of phosphorous and nitrogen, causing the incidence of fish death due to the toxic algae blooms or by the oxygen lack caused by algae decomposition^42^.

**Figure 12:**
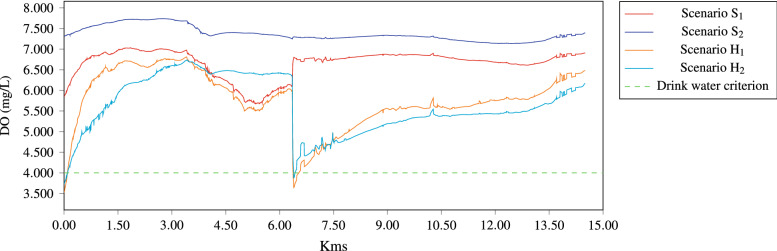
Concentration of DO, along the river when the hydrodinamic is steady.

For dissolved oxygen, scenarios S$_1$ and S$_2$ satisfy the ecological criteria for aquatic life and drinking water protection. Likewise, H$_1$ and H$_2$ scenarios showed that even when dissolved oxygen concentrations reach values below 4 mg/L, the river can recover oxygen levels in a relatively short distance. Dissolved oxygen, in H$_1$ were lower than concentrations obtained for H$_2$, in H$_1$ season dissolved oxygen, ranges from 6.5 to 7.4 mg/L, meanwhile, H$_2$ the values range from 7.1 to 7.8 mg/L (see Fig. 12). The behavior of the dissolved oxygen is correlated to the temperature, increases DO concentrations, and when the latter increases dissolved oxygen decreases due to increased solubility and additionally biochemical processes and biological metabolisms consume more dissolved oxygen. Similarly, other studies state that dissolved oxygen is temperature-dependent, since warmer waters are capable of dissolving smaller amounts of oxygen, hence, a hot water discharge can decrease the dissolved oxygen to levels below the limit necessary for some types of aquatic life.

Although the Gallinas River provides adequate dissolved oxygen concentrations for the vast majority of fishes and other aquatic organisms, this parameter can not be evaluated in terms of assimilation capacity, because it does not act as a pollutant, nevertheless, is a very important water quality parameter interconnected with nitrification and phosphorus cycle processes. When the dissolved oxygen decreases affect the phosphate concentrations leading to algae proliferation, in fact, 1 g of phosphate causes the growth of approximately 100 g of algae. The algae decomposition processes can generate oxygen consumption of around 150 g. In addition to the foregoing oxygen consumption is affected by nitrification processes.

According to the simulations results, a clear cause–effect correlation can be observed. This correlation is mainly a function of the increase or decrease of water flow in the river, the distance traveled, the type of pollutant, and the surface or groundwater inputs from the surrounding sites.

Increases in flow, distance traveled and runoff from sites with high fertilization and application of agrochemicals with high phosphorus content, allow greater entrainment of PO4-P into surface streams. Due to the low solubility of PO4-P and its high presence in the sites, a higher concentration occurs as the flow increases.

In the case of NH3-N, it was observed that there is a dilution effect as flows increase, i.e., the higher the flow, the lower the concentration. This may be due to the high mobility and solubility of the pollutant, while ionization, nitrification, and denitrification conditions of the compound occur.

With respect to COD, it was observed that there is a broad and inversely proportional correlation with the flow. As the flow increases, as long as there are no additional punctual or diffuse inputs, the concentration tends to decrease due to a dilution effect.

In the case of the DO, it was observed that it is highly sensitive to the natural aeration conditions resulting from the topographic and morphological conditions of the river, the results show that, due to the presence of waterfalls, the DO increases considerably.

## Conclusions

Today assimilation capacity is considered one of the many benefits of ecosystem health and integrity, therefore, this paper determined the assimilation capacity when river pollution is controllable, under two characteristics scenarios and two hypothetical.

The simulation of the pollution movement in the river was based on the mathematical equations of pollution transport, implemented in the EFDC model. The procedure was applied to the Gallinas River in Mexico and all the obtained results were presented in terms of the concentration along the river of the phosphorus, nitrate, and dissolved oxygen.

This is one of the first studies in the area and serves as a starting point for environmental authorities and decision-makers to implement plans, programs, and projects aimed at reducing and controlling water pollution, seeking to reduce nutrient loads on the Gallinas River. It also manages to generate knowledge in the surrounding communities about the care and preservation of water resources, since the river provides an important supply of water for the socio-economic activities of the region.

Determine numerically rivers self-purification capacity for pollution treatment can bean effective and low-cost method. There are numerous examples of pollutant discharges to rivers that have caused irreparable damages to plant and animal species, and posed risks to humans. Therefore, assessing a rivers capacity to assimilate pollutants through adjustment of its flow is an important water-quality management tool.

Numerical simulations carried out with the EFDC model presented above were performed for wet and dry seasons, and additionally two hypothetical scenarios. According to the results, was evidenced that PO$_4$-P contamination in the Gallinas River is more important in the wet season since it presented concentrations that exceed the ecological criteria for the use of drinking water and protection of aquatic life (0.1 mg/L) throughout the section of the study. For the dry season, this pollutant becomes representative once the Tamasopo river is taxed, which means that the Tamasopo river is a relevant tributary in the contribution of PO$_4$-P. To control PO$_4$-P contamination in the Gallinas river, it is necessary to reduce the concentration by up to 80% in the Piedritas and Tamasopo tributaries, both in the dry and wet season, thus, the PO$_4$-P levels would be maintained below the ecological criteria.

As for the NO$_3$-N quality determinant, the Gallinas river is capable of assimilating the pollutant both in the dry and wet seasons, only if the concentrations of external discharges (Piedritas stream and Tamasopo river) around a 300%, would exceed the allowable value in dry weather.

Dissolved oxygen presented an adequate behavior trend in the two seasons, however, it is clear that the concentrations were lower in summer and this is due to the direct influence that the increase in temperature has on said variable. Regarding the approach of scenarios H$_1$ and H$_2$, it is inferred that the Gallinas river has an excellent reaeration capacity, because although the concentrations were reduced by up to 50% (3 mg DO/L), the results showed that the river recovers DO satisfactorily.

Also can be inferred, according to the simulations, during the rainy season for the hypothetical scenario, can occur an increase of the PO$_4$-P concentration compared to the dry season, due to the leaching of the fertilizers present on the sown surface.

Tamasopo river is the most influential source for the Gallinas river, both in terms of flow and pollutants, since it was found that once the Tamasopo river joins the Gallinas river, the concentrations of the variables analyzed in the study area are seen affected by said contribution, concluding that the source in question is decisive in the quality of the Gallinas river water.

It is important to bear in mind that there are diffuse sources that also influence the physicochemical characteristics of water bodies, therefore, it is not enough to affirm that the study area can be effected only by tributaries: Piedritas stream and Tamasopo river, which were the ones that could be seen with the naked eye. Nevertheless, it can be concluded that there is a clear correlation between the punctual discharges of various chemical products to the Tamasopo river that converges in turn with the Gallinas river, as well as the diffuse discharges, a product of the leaching of soils with intensive agriculture in the area of influence of the Gallinas River.

EFDC Explorer model was efficient for the purposes of this research, it allows to generate curvilinear grids that adjust in detail to the conditions of the body of water, demonstrating that the model well-calibrated can reproduce with high accuracy the contaminants transport reality.

Finally, the results of the simulations for the hypothetical conditions, shows that the river could has a good assimilation capacity, despite, the increase in concentrations and additionally, the following policies and actions could be proposed to prevent, control, and improve conditions in the Gallinas River sub-basin.

- Continue with a periodic and systematic monitoring plan along the Gallinas River, with flow measurements, water quality parameters in situ, and taking samples for parameter analysis, in order to establish immediate measures when parameters outside the norm or ecological criteria are detected.
- Establish permanent monitoring of the effluents of the Alianza popular sugar mill, which contributes its wastewater to the Tamasopo River, which is, in turn, the most important tributary of the Gallinas River. It is suggested to carry out a general inspection, to verify the optimal wastewater treatment plant, and to carry out periodic inspections to determine if these effluents are adequately treated before being incorporated into the river Tamasopo
- Surrounding urban populations such that have drainage and wastewater treatment plants must be supervised so that such plants are in operation and their effluents comply with the respective standards.
- For the cases of homes that do not have piped water systems, and that obtain water for their domestic use from the Gallinas River through motor pumps, it is recommended to warn to the population, through the corresponding health authorities, on the need to make water drinkable by the different known means.
- In the case of diffuse contamination by runoff of agrochemicals that end up in the riverbed, it is important to link up with the federal and state government agencies, so that an extension program is implemented and the optimal doses are recommended of fertilizers for crops, as well as the most efficient irrigation systems to apply irrigation water and essential nutrients for the optimal development of the crop.
- The interaction between civil society (companies, inhabitants and producers), the federal, state and municipal governments, it is essential for the Gallinas river sub-basin to be a place where biotic and abiotic resources (water, soil, fauna and flora), as well as the people who inhabit it, they are under an environmentally sustainable system.
